# Supplementary material for: A primary sensory cortical interareal feedforward inhibitory circuit for tacto-visual integration
Source: Nat Commun. 2024 Apr 10;15:3081. doi: 10.1038/s41467-024-47459-2 (PMC11003985; doi:10.1038/s41467-024-47459-2)
Supplement: Supplementary file 1 — Supplementary Information [file 41467_2024_47459_MOESM1_ESM.pdf]

A primary sensory cortical interareal feedforward inhibitory circuit for tacto-visual integration

Weiler et al.

Supplementary information

Supplementary Figures 1-7

Supplementary Table 1

This file contains:

Supplementary Figures 1-7
Supplementary Table 1
Supplementary References

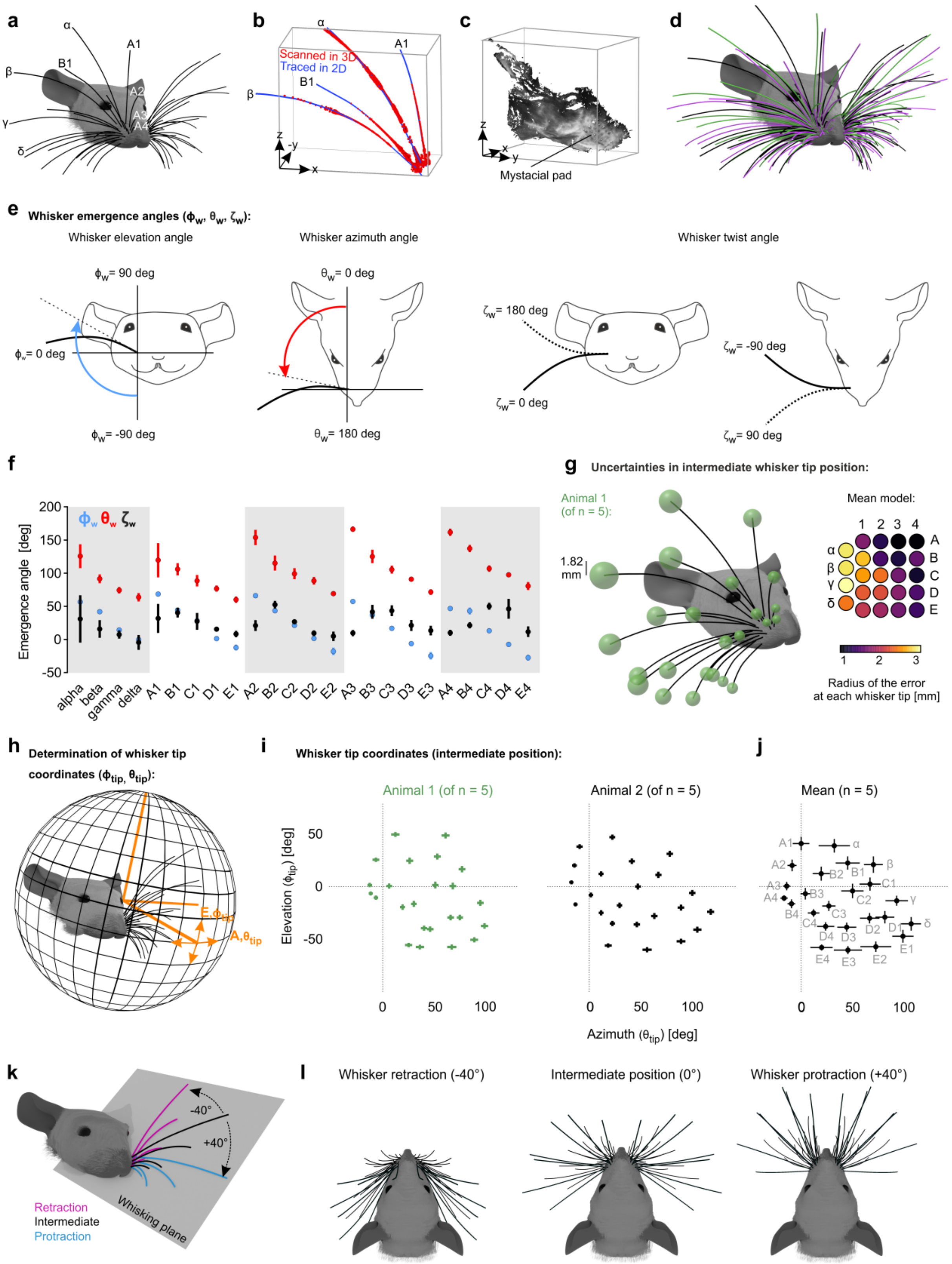

Supplementary Fig. 1: **Reconstruction of the mouse whisker array.** (**a**) Representative reconstructed 3D model of the mouse whisker array aligned with a realistic 3D model of the mouse head (Bolanos et al., 2021) [1]. Letters indicate the standard nomenclature for the labeled whiskers. (**b**) After their 3D-reconstruction, all whiskers were plucked, traced in 2D and aligned to their corresponding 3D point clouds. (**c**) 3D point cloud of the mouse head including the mystacial whisker pad (obtained after whisker plucking). (**d**) Morphologically accurate model of the mouse head together with 3D-reconstructed superimposed whiskers from three different exemplary mice. (**e**) Schematic illustration shows how emergence angles were determined. In the final reconstructed models of the whisker arrays, we determined the emergence angles (ϕ_w, θ_w and ζ_w) of each whisker (5x24 whiskers in total). (**f**) Quantification of average emergence angles ± s.e.m. Note, that s.e.m. accounts for the deviation in the

biological data (n=5 mice). The horizontal plane was defined to be parallel to the bregma-lambda plane. (**g**) Left: Representative reconstructed whisker array. The green spheres on the tip of each whisker represent uncertainties in the whisker tip positions caused by measurement uncertainties in the emergence angles and whisker basepoint positions (see Methods: Error estimation in whisker tip position). Note that the given scale bar only accounts for the sphere of the whisker next to it, as this is a 3D illustration. Right: Color-coded map of the whisker tip uncertainties of the average whisker array (of n=5 mice). Given radii include both measurement uncertainties in individual mice and the deviation of biological data. (**h**) A left eye-centered spherical coordinate system was used to determine the position of each whisker tip (ϕ_{tip} and θ_{tip}) in elevation (E) and azimuth (A) with respect to the eye. The horizontal plane was defined to be parallel to the bregma-lamda plane. (**i**) Representative elevation and azimuth coordinates of all 24 large whisker tips with respect to the left eye (from 2 out of 5 mice). Note, that the error bars represent measurement uncertainties in the emergence angles and basepoint locations (see Methods). (**j**) Average elevation and azimuth coordinates of all whisker tips (n=5 mice). Error bars account for both measurement uncertainties in individual mice and the deviation of biological data. Letters indicate the standard nomenclature of the 24 large whiskers. (**k**) For simulation of whisker retraction and protraction, whiskers of each row were rotated around their base point to -40° or +40° along a whisking plane fitted through their base points and tips. (**l**) Representative examples of whisker retraction (-40°), the intermediate position (0°) and whisker protraction (+40°). Source data are provided as a Source Data file.

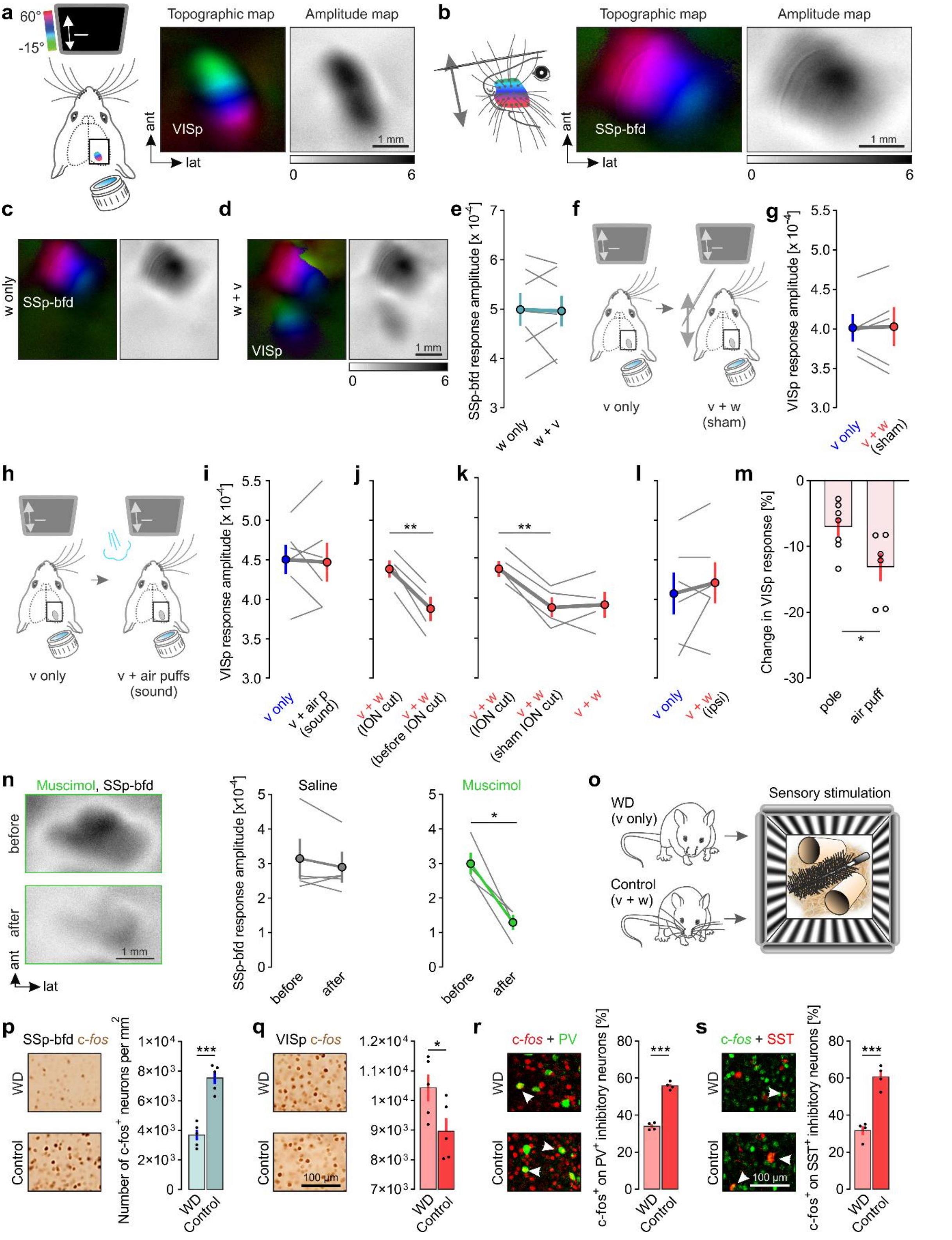

Supplementary Fig. 2: Whisker stimulation suppresses visually driven activity in VISp in vivo. Related to Fig. 2. (**a**, **b**) Schematics of unimodal visual or whisker stimulation procedures together with the obtained topographic and amplitude maps of VISp and SSp-bfd. (**c, d**) Cortical topographic and amplitude maps evoked after whisker stimulation alone (w only) and bimodal whisker and visual stimulation (w+v). (**e**) Quantification of SSp-bfd response amplitudes under w only and w+v conditions (n=7 mice). (**f**) Schematic of unimodal visual and sham whisker stimulation after whisker deprivation (WD). (**g**) Quantification of VISp response amplitudes (n=5, p=0.8744; paired t-test). (**h**) Schematic of stimulation procedures to test for the influence of the sounds created by the air puffs on visually driven VISp activity. In the bimodal stimulation condition, air puffs were directed to the same space as for whisker stimulation, however, after the whiskers were removed. (**i**) Quantification of VISp response amplitudes evoked under the stimulus conditions depicted in (**h**) (n=6 mice, p=0.8173; paired t-test). (**j**) Quantification of VISp response amplitudes evoked under bimodal stimulation (v+w; w: air puffs) before and after ION cut

(n= 4 mice, p=0.0049; paired t-test). (**k**) Quantification of VISp responses evoked under bimodal stimulation conditions (v+w; w: air puffs) before and after a sham ION cut and after finally cutting the ION (n=4 mice, p=0.0368, p=1; paired t-test followed by Bonferroni correction). Sham ION cut was performed by opening the skin above the ION and touching the nerve gently with fine forceps. (**l**) Quantification of VISp responses evoked under v only and v+w stimulation conditions. Here, whiskers ipsilateral to the recorded hemisphere were stimulated using air puffs. (n=6 mice, p=0.3742; paired t-test). (**m**) Percentage change of visually evoked VISp activity by simultaneous whisker stimulation with either the pole or air puffs (n=7 and n=6 mice, p=0.03; unpaired t-test). Bars represent means ± s.e.m. (**n**) Left: Representative amplitude maps of SSp-bfd obtained after air puff stimulation of the whiskers before and after muscimol injections in SSp-bfd. Right: Whisker stimulation evoked SSp-bfd amplitudes before and after saline or muscimol injections (saline, p=0.4623; muscimol, p=0.0195; paired t-tests). (**o**) Schematic of procedures preceding c-fos immunohistochemistry. Awake control and WD mice were placed in an environment for multisensory stimulation. (**p**) Left: Representative images of c-fos expression in SSp-bfd in WD and control mice after their exposure to the enriched environment. Right: Quantified density of c-fos labeled neurons in SSp-bfd (n=5 mice per group, p=0.0001; unpaired t-test). (**q**) Left: Representative images of c-fos expression in VISp in WD and control mice after exposure to the same environment. Right: Density of c-fos labeled neurons in VISp (n=5 mice per group, p=0.0490, unpaired t-test). (**r,s**) Left: Representative images of double immunostainings against c-fos and either PV or SST positive interneurons in VISp in WD and control mice after exposure to the same environment. Right: Percentage of PV or SST positive interneurons double labeled with c-fos of the total number of PV or SST positive interneurons (n=4 mice per group, p=0.0000, p=0.0000; unpaired t-test). Solid grey lines connect measurements of individual mice and circles indicate means ± s.e.m. Bars always represents means ± s.e.m. *p<0.05, **p<0.01, ***p<0.01. Source data are provided as a Source Data file.

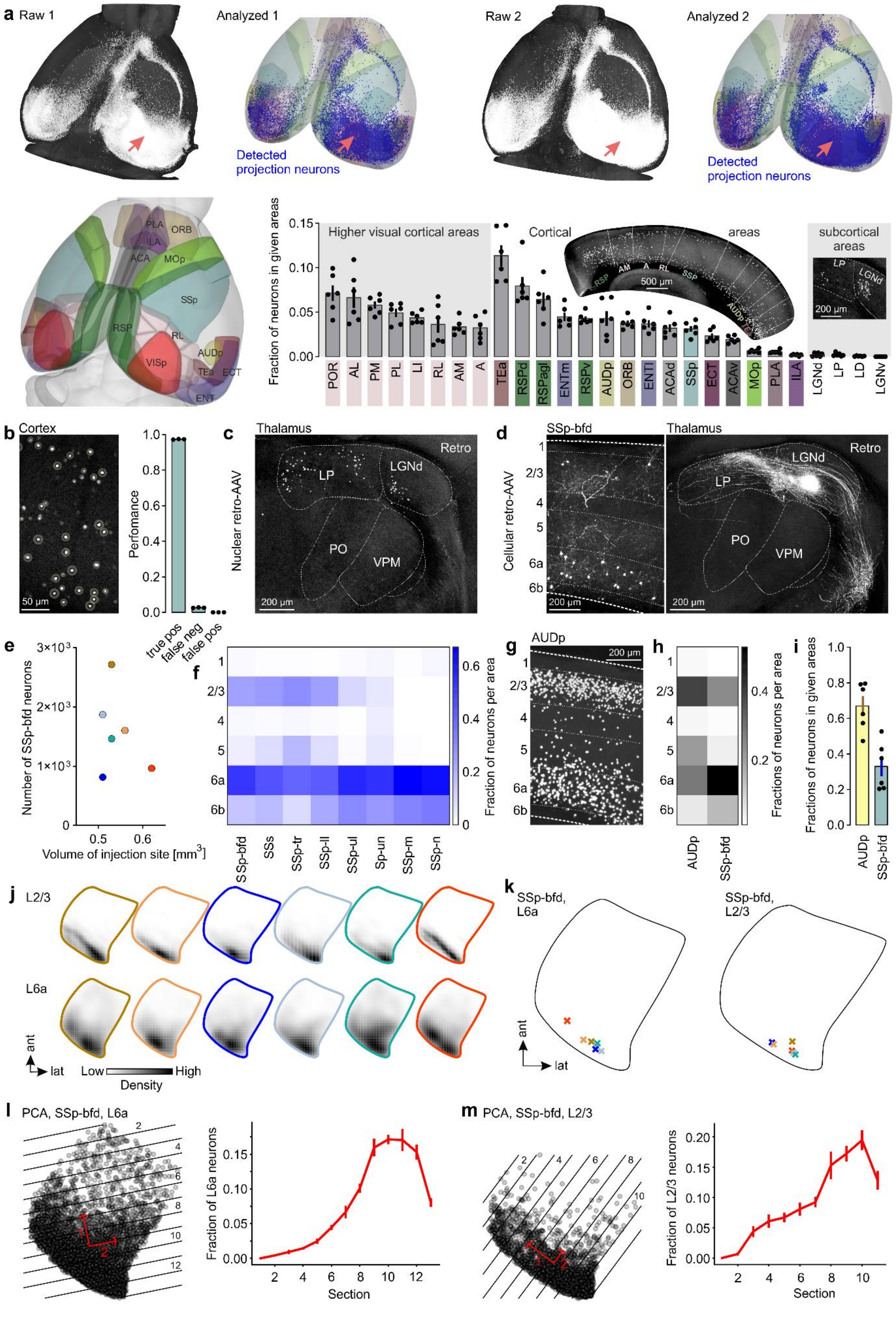

Supplementary Fig. 3: Analysis of the distribution of neurons projecting to VISp. (**a**) Top: Representative 3D mouse brains reconstructed from individual slices imaged by two-photon tomography of retro-AAV injected (into VISp, labeled neurons: white) brains together with 3D rendered mouse brains showing the neurons detected using cellfinder [2] (blue, shown

is every 10th detected neuron, two from six brains in total are depicted). Bottom, left: 3D rendered mouse brain with colored cortical brain areas containing VISp projecting neurons. Bottom, right: Fraction of projection neurons in different ipsilateral brain areas. Black circles indicate fractional cell counts of individual mice (n=6 mice). Bars represent means ± s.e.m. (**b**) Left: Retrogradely labeled neurons in the mouse cortex. Neurons correctly detected by cellfinder [2] are marked by a ring. Right: Performance of cellfinder in detecting labeled neurons correctly. Black circles indicate fractional cell counts of individual mice (n=3). Bars represent means. (**c, d**) Example images showing neurons in thalamic areas and SSp-bfd retrogradely labeled by either the nuclear or the cellular retro-AAV (AAV-EF1a-H2B-EGFP, rAAV2-retro.CAG.GFP) after their injection into VISp. Numbers in (**d**) indicate cortical layers. (**e**) The absolute number of detected projection neurons in SSp-bfd from six different mice was plotted against the volume of the injection site in VISp (r=-0.3115, p=0.5478). (**f**) Color-coded average fraction of projection neurons in cortical layers across somatosensory cortical areas (n=6 mice). Numbers on the left indicate cortical layers. Values are normalized to the maximum within each area. (**g**) Representative coronal section showing retrogradely labeled neurons in AUDp. Numbers indicate cortical layers. (**h**) Grey-scale-coded average fraction of projection neurons in cortical layers of AUDp and SSp-bfd in the same mice (n=6). Numbers indicate cortical layers. Values are normalized to the maximum within each area. (**i**) Fraction of projection neurons in AUDp and SSp-bfd. Black circles indicate fractional cell counts of individual mice (n=6). Bars represent means ± s.e.m. (**j**) Density maps of projection neurons in L6 and L2/3 of SSp-bfd (horizontal projection) of six individual mice. Border colors indicate the corresponding injection sites in VISp (see Fig. 3i). (**k**) Horizontal projections of L6 and L2/3 of SSp-bfd. Crosses indicate the location of the maximum density of the six different projections neuron populations. Colors relate to the different injection sites in VISp (see Fig. 3i). (**l, m**) Left: horizontal projections of L6 and L2/3 of SSp-bfd. Grey circles indicate projections neurons labeled by all six injection sites in VISp. Red arrows indicate the first and second principal components determining the direction of the spatial dispersion of projection neurons. Black lines: Section borders orientated vertically to the first principle component. Right: The average fraction of projection neurons in all sections of L6 and L2/3 (n=6). PCA: Principal component analysis. Source data are provided as a Source Data file.

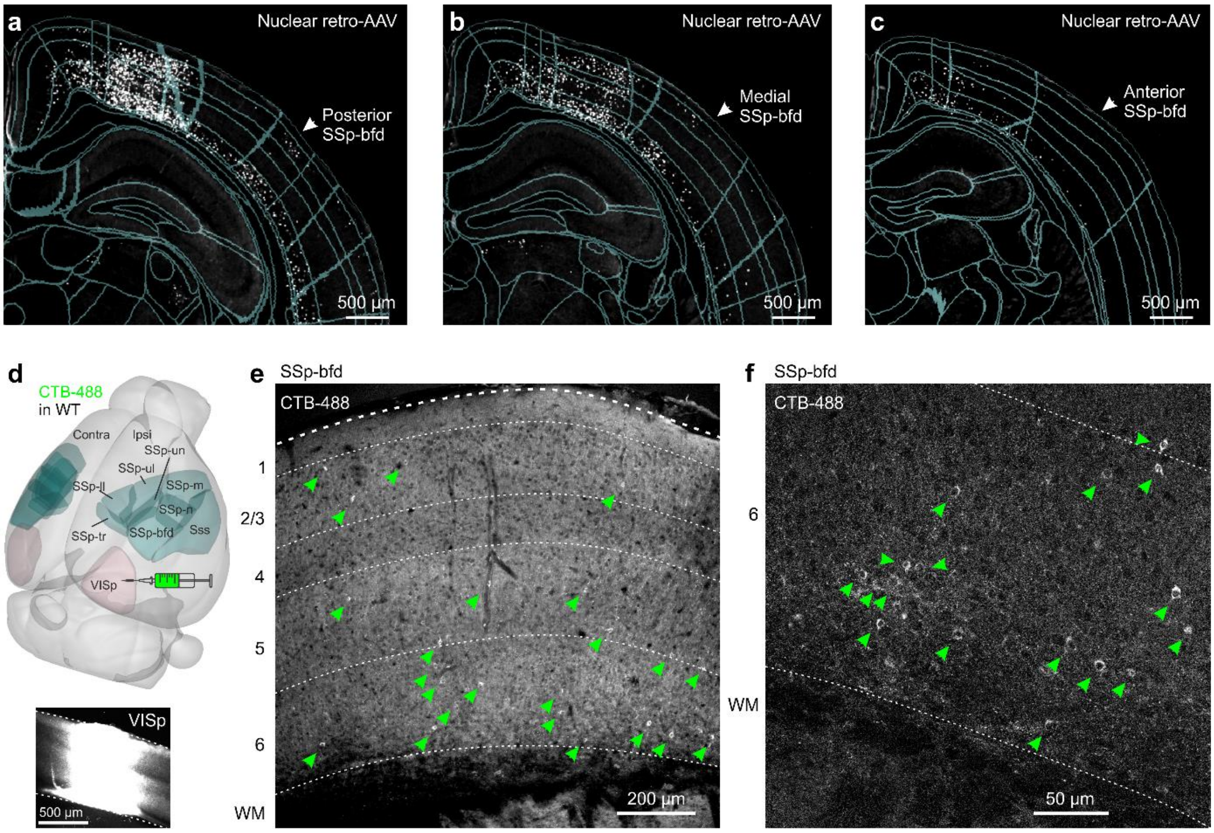

Supplementary Fig. 4: Layer 6 in SSp-bfd is the main source for direct connnections to VISp. (**a**-**c**) Representative coronal sections showing retrogradely labeled VISp projecting neurons in the posterior, medial and anterior SSp-bfd and in other brain areas. Turquoise lines indicate areal borders and cortical layers. (**d**) Top: 3D-rendered mouse brain showing the locations of VISp and somatosensory cortical areas (including SSp-bfd) together with a schematic of the choleratoxin subunit B (CTB) injection approach. Bottom: Coronal section showing a representative injection site of CTB in VISp. (**e**) Representative coronal sections of SSp-bfd with CTB labeled VISp projecting neurons. Numbers indicate cortical layers and green arrows indicate locations of labeled cells (n=1 mice). (**f**) Representative CTB labeled VISp projecting neurons in L6 of SSp-bfd highlighted with green arrows.

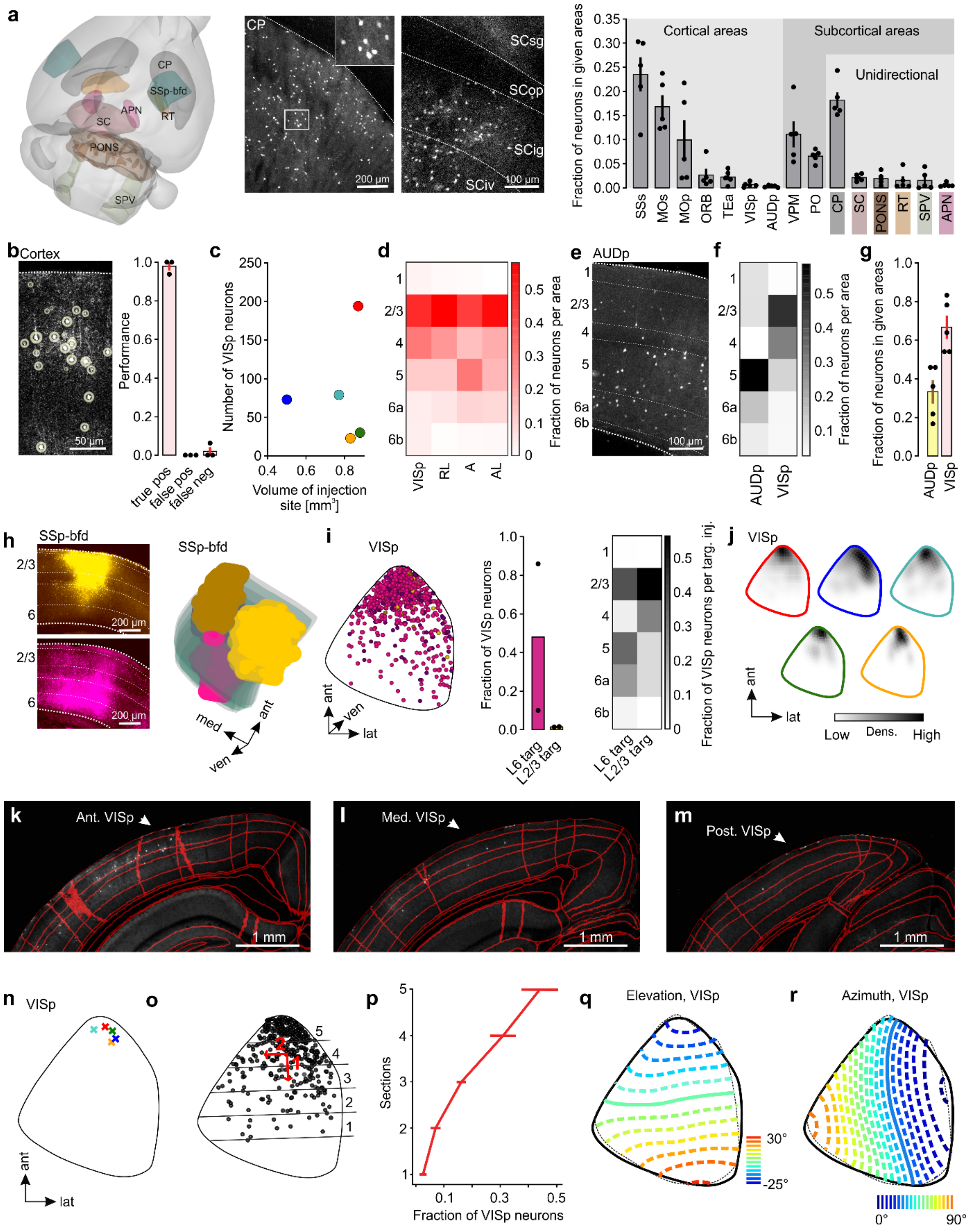

Supplementary Fig. 5: Related to Fig. 5. (**a**) Left: 3D-rendered mouse brain with colored subcortical brain areas unidirectionally targeted by SSp-bfd. Middle: Representative coronal images of postsynaptic neurons in the caudoputamen (CP) and superior colliculus (SC). Right: Fraction of postsynaptic neurons in different cortical and subcortical brain areas. Black dots indicate fractional cell counts of individual mice (n=5). Bars indicate means ± s.e.m. (**b**) Left: Anterogradely labeled neurons in the mouse cortex. Neurons correctly classified by the cellfinder software [2] are marked by a ring. Right: performance of cellfinder in detecting labeled neurons correctly (n=3 mice). (**c**) The absolute number of detected postsynaptic neurons in VISp from five different mice was plotted against the volume of the corresponding injection site in SSp-bfd (r=0.1028, p=0.8694). (**d**) Color-coded average fraction of postsynaptic neurons across cortical layers in visual cortex areas (n=5 mice). Numbers on the left indicate cortical layers. Values are normalized to the maximum within each area. (**e**) Representative coronal image showing postsynaptic neurons in AUDp (white). (**f**) Grey-scale-coded average fraction of postsynaptic neurons across cortical layers of AUDp in comparison to VISp (n=5). Numbers indicate cortical layers. Values are normalized to the maximum within each area. (**g**) Fraction of postsynaptic neurons in AUDp and VISp. Black dots indicate fractional cell counts of

individual mice (n=5). Bars indicate means ± s.e.m. Values are normalized to the maximum within each area. (**h**) Left: Coronal images showing representative injection sites targeted to L2/3 and L6 in SSp-bfd. Right: 3D-reconstructions of L2/3 and L6 targeted injection sites warped into the 3D-rendered space of SSp-bfd of the CCFv3 [3]. Yellow colors indicate L2/3 targeted and purple colors indicate L6 targeted injection sites (n=2 mice for each condition). (**i**) Left: horizontal projection of VISp. Postsynaptic neurons labeled by both L2/3 and L6 targeted injections were warped to VISp of the CCFv3. Middle: Fraction of postsynaptic neurons in VISp labeled by either L2/3 or L6 targeted injections to SSp-bfd. Right: Average fraction of postsynaptic neurons in different cortical layers (numbers) of VISp labeled by either L2/3 or L6 targeted injections to SSp-bfd. Numbers indicate cortical layers. Values are normalized to the maximum obtained after L6 or L2/3 targeted injections, respectively. (**j**) Density map of postsynaptic neurons in VISp (horizontal projection) of five individual mice. Border colors indicate the corresponding injection sites in VISp (see Fig. 4f). (**k-m**) Representative coronal sections showing anterogradely labeled postsynaptic neurons in the anterior, medial and posterior part of VISp as well as in neighboring cortical areas. Red lines indicate areal borders and cortical layers. (**n**) Horizontal projection to VISp. Crosses indicate the maxima of density of the five different populations of projections neuron. Colors correspond to the different injection sites in VISp. (**o**) Horizontal projection of VISp. Grey circles indicate postsynaptic neurons labeled by all five injection sites in VISp. Red arrows indicate the first and second principal components determining the direction of spatial dispersion of neuron locations. Black lines are section borders orientated vertically to the first principal component. (**p**) Average fraction of projection neurons was counted in all sections of VISp. (**q,r**) Elevation and azimuth contour plots of VISp obtained by GCaMp6 fluorescence-based visuotopic imaging [4]. Dashed black line: mean field sign borders of VISp. Solid black line: horizontal projection of the areal border of VISp from the CCFv3. Solid colored lines represent the horizontal meridian. Individual contours are 5° apart (modified from (Zhuang et al., 2017) [4]). Source data are provided as a Source Data file.

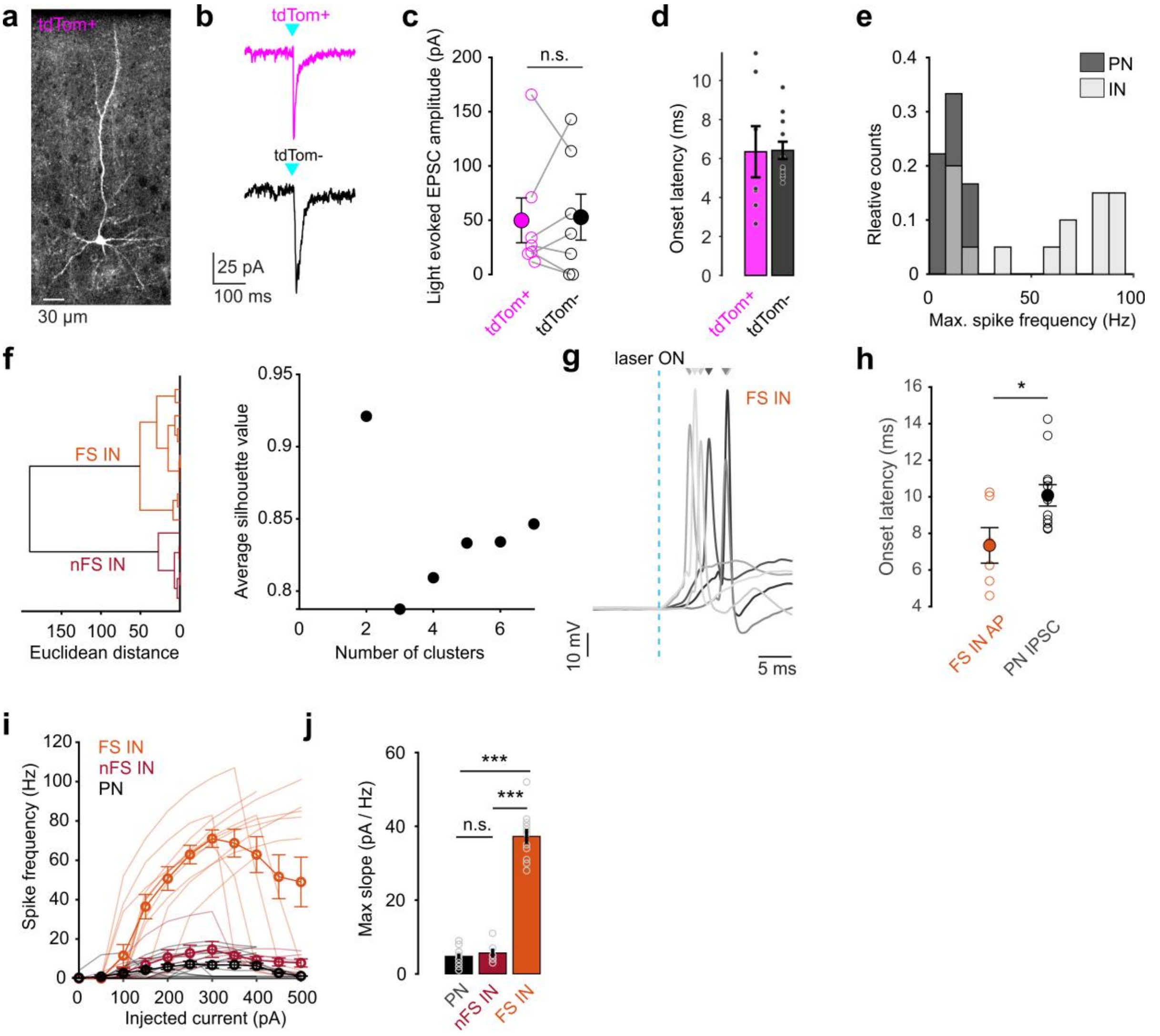

Supplementary Fig. 6: SSp-bfd mediates feedforward inhibition onto L2/3 neurons in V1. (**a**) Anterogradely labelled L2/3 cell (tdTom^{+}) in VISp following AAV.hSyn.Cre injection in SSp-bfd of Ai14 mice. Scale bar: 30 µm. (**b**) Representative example of light-evoked EPSC in a tdTom^{+} and a tdTom^{-} L2/3 cell. Scale bars: 25 pA, 100 ms. (**c**) Comparison of evoked peak EPSCs for tdTom^{+} and a tdTom^{-} cells. Mean (filled circles) and individual data points (empty circles) are displayed. Lines connect neighboring cells (<100 µm apart; n=7 cells from five mice). Error bars are s.e.m. (p=0.87, Wilcoxon rank-sum). (**d**) Onset latency of light-evoked EPSCs for tdTom^{+} and a tdTom^{-} (n=6 and 13 cells from six mice). Error bars are s.e.m. (p=0.15, Wilcoxon rank-sum). (**e**) Distribution of maximal spike frequency for excitatory PNs and inhibitory interneurons (PN, IN; n=13 and 17 cells). Maximal spike frequency was obtained using current injections. (**f**) Left: Dendrogram of unsupervised hierarchical cluster analysis using Ward's method showing two major groups of interneurons based on their maximal spike frequency. Right: Average silhouette values plotted against cluster number. Note that the silhouette value is highest for two clusters. (**g**) Action potentials in FS INs evoked by SSp-bfd stimulation (n=6 cells). Arrow heads indicate peak of action potentials. Scale bar: 5 ms (**h**) Onset latencies for light-evoked action potential peak of FS INs and onset latencies of light-evoked IPSCs in excitatory PNs (n=6 and n=12 cells from six mice, p=0.0039, Wilcoxon rank-sum). (**i**) Maximal spike frequency plotted against injected current amplitude for PNs, nFS INs and FS Ins (n=13, 6 and 11 cells from seven mice). Mean (thick lines with circles) and individual data (thin lines) are displayed. (**j**) Maximal slopes for curves in (**i**) for PNs, nFS INs and FS INs. Mean and individual data points are displayed. Error bars are s.e.m. (p<0.001, Kruskal-Wallis). Source data are provided as a Source Data file.

Supplementary Table 1. Parameter values. Table lists the default parameter values used for simulating the basal network model. Eqs. (1-3) were used to simulate the model. Eqs. (4-6) were obtained by deriving the analytical solutions. Previous studies refer to [5] and [6]. All parameter values were set according this table, unless otherwise specified, as follows. **Fig. 7b** (inset): $I_P^V = I_F^V = 0$. **Fig. 7c**: I^{cm} was varied. With threshold: $I^{cm} \leftarrow \min\left(I^{cm}, \rho\right)$. Without threshold: $I^{cm} \leftarrow I^{cm}$, i.e. $\rho \to \infty$. Left, $I^{cm} = 1$. **Fig. 7d** (left, dashed lines): $\theta_P = 0.6$. **Fig. 7e**: α, and accordingly J_{PF}, were varied ($J_{PF} = \alpha \times J_{PP}$). Left (dashed lines): $\alpha = 2.5$, $J_{PF} = 5$. **Fig. 7f**: G_P and/or G_F were varied. Left (dashed lines): $G_F = 8$.

<table>
<tr><th colspan="2">Parameter names</th><th>Parameter values</th><th>In consistent with</th><th>Relevant equations</th></tr>
<tr><td colspan="2">Population firing gain</td><td>$G_P = 1$
$G_F = 5 \times G_P = 5$</td><td>Supplementary Fig. 6i-j</td><td>Eqs. (3-6)</td></tr>
<tr><td colspan="2">Population activation-threshold</td><td>$\theta_P = 0.5$
$\theta_F = 0.7 \times \theta_P = 0.35$</td><td>Fig. 6o</td><td>Eqs. (2-5)</td></tr>
<tr><td colspan="2">Population activity time constant</td><td>$\tau_P = 60$ ms
$\tau_P = 12$ ms</td><td>Previous studies</td><td>Eqs. (1,4), and ISN criteria</td></tr>
<tr><td colspan="2">Threshold of I^{cm} postsynaptic strength</td><td>$\rho = \delta\theta_P = 0.5$
$(\delta = 1, \rho \geq 0)$</td><td>Fig. 6i-j</td><td>Eqs. (2,4,5)</td></tr>
<tr><td colspan="2">Cross-modal input</td><td>$I_P^{cm} = I_F^{cm} = I^{cm} = 0.6$
(initial value; note Eq. 2)</td><td>Fig. 6l-m</td><td>Eqs. (1,2,4,5)</td></tr>
<tr><td colspan="2">I/E ratio on PN population</td><td>$\alpha = 1/\left(\text{EI ratio}\right) = 2$</td><td>Fig. 6f-g</td><td>Eqs. (1,5,6)</td></tr>
<tr><td colspan="2">Visually-driven input</td><td>$I_P^V = 3.6$
$I_F^V = 0.55$</td><td>Previous studies</td><td>Eqs. (1,4)</td></tr>
<tr><td rowspan="4">Absolute (J_{ij}) and effective (w_{ij}) synaptic weights</td><td>PN → PN</td><td>$J_{PP} = 2$
($w_{PP} = G_P \times J_{PP} = 2$)</td><td>Previous studies</td><td rowspan="4">Eqs. (1,4-6)</td></tr>
<tr><td>FS → PN</td><td>$J_{PF} = \alpha \times J_{PP} = 4$
($w_{PF} = G_P J_{PF} = 4$)</td><td>Fig. 6f-g</td></tr>
<tr><td>FS → FS</td><td>$J_{FF} = 0.8$
($w_{FF} = G_F J_{FF} = 4$)</td><td>Previous studies</td></tr>
<tr><td>PN → FS</td><td>$J_{FP} = 0.4$
($w_{FP} = G_F J_{FP} = 2$)</td><td>Previous studies</td></tr>
</table>

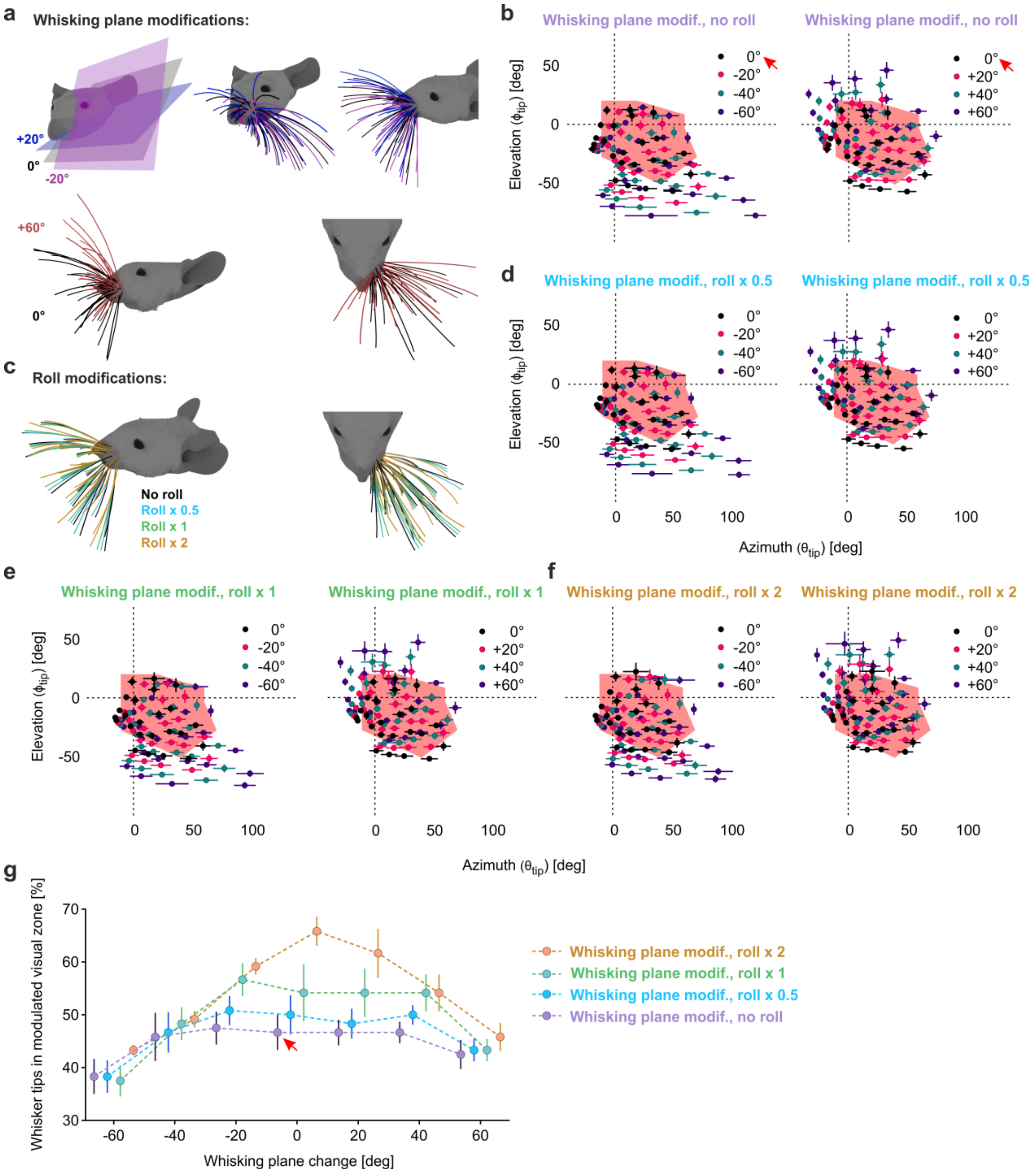

Supplementary Fig. 7: Analysis of the overlap of the modulated visual space with whisker tips after computationally simulating multiple whisker trajectories during protraction. (**a**) Upper row: Schematics of the simulation of alternative whisking planes on the example of the C-row of whiskers. Whisking planes were gradually modified in 20° steps from -60° to +60°. Note, that each row of whiskers has an individual whisking plane which was also adjusted individually (see Methods).The black whiskers display the whisker array protracted along the 0° whisking plane and the blue and magenta whiskers show the same whisker array protracted along the +20° and -20° whisking plane, respectively. Lower row: Representative whisker positions in the same whisker array in 3D after protraction caused by adjusting the whisking plane from 0° (black) to +60° (red). Note that at +60° some (red) whiskers cross the midline of the mouse in front of the snout, suggesting unrealistic whisking trajectories during protration. (**b**) Average elevation and azimuth (ϕ_{tip} and θ_{tip}, respectively) coordinates of whisker tips after adjusting the whisking plane from -60° to 0° (left) and from 0° to +60°. Errors represent the s.e.m. (n=5 mice). The bright pink area indicates the modulated visual space covered by VISp extended by eye movements in a ± 20° range. The red arrow indicates the whisking model used in Fig. 1 and Fig. 8 (main model) (**c**) Representative example for the simulations of different "roll" angles (see Methods: Simulation of alternative whisking trajectories). (**d-f**) Average elevation and azimuth coordinates of whisker tips (ϕ_{tip} and θ_{tip}, respectively) after combining simulated adjustments in the whisking planes and "roll" angles. Errors represent the s.e.m. (n=5 mice). The bright pink area indicates the modulated visual space covered by VISp extended by eye movements in a ± 20° range. (**g**) Quantification of the percentage of whisker tips in the modulated visual space under the different simulations of whisker trajectories during protraction. Error bars indicate the s.e.m. (n=5 mice). The red arrow

indicates the whisking model used in Fig. 1 and Fig. 8 (main model). Note, that the error bars presented in **b-f** do not include measurement uncertainties in individual mice, as their effect on the final tip coordinates was negligible. Source data are provided as a Source Data file.

Supplementary References

1 Bolanos, L. A. *et al.* A three-dimensional virtual mouse generates synthetic training data for behavioral analysis. *Nat Methods* **18**, 378-381, doi:10.1038/s41592-021-01103-9 (2021).

2 Tyson, A. L. *et al.* A deep learning algorithm for 3D cell detection in whole mouse brain image datasets. *Plos Computational Biology* **17**, doi:10.1371/journal.pcbi.1009074 (2021).

3 Wang, Q. X. *et al.* The Allen Mouse Brain Common Coordinate Framework: A 3D Reference Atlas. *Cell* **181**, 936-+, doi:10.1016/j.cell.2020.04.007 (2020).

4 Zhuang, J. *et al.* An extended retinotopic map of mouse cortex. *Elife* **6**, doi:10.7554/eLife.18372 (2017).

5 Ozeki, H., Finn, I. M., Schaffer, E. S., Miller, K. D. & Ferster, D. Inhibitory stabilization of the cortical network underlies visual surround suppression. *Neuron* **62**, 578-592, doi:10.1016/j.neuron.2009.03.028 (2009).

6 Murphy, B. K. & Miller, K. D. Balanced amplification: a new mechanism of selective amplification of neural activity patterns. *Neuron* **61**, 635-648, doi:10.1016/j.neuron.2009.02.005 (2009).
