# Supplementary material for: A primary sensory cortical interareal feedforward inhibitory circuit for tacto-visual integration
Source: Nat Commun. 2024 Apr 10;15:3081. doi: 10.1038/s41467-024-47459-2 (PMC11003985; doi:10.1038/s41467-024-47459-2)
Supplement: Supplementary file 3 — Description of additional supplementary files [file 41467_2024_47459_MOESM3_ESM.pdf]

DESCRIPTION OF ADDITIONAL SUPPLEMENTARY FILES DOCUMENT

Supplementary Movie 1: 3D reconstructed mouse whisker array overlaid with the visual space covered by the primary visual cortex (VISp, bright red space). Whiskers were aligned to a realistic model of the mouse head (Bolanos et al., 2021) and the visual space was constructed based on data from Zhang et al., 1017.

Supplementary Movie 2: Simulated whisker retraction (-40°) and protraction (+40°). Whiskers were aligned to a realistic model of the mouse head (Bolanos et al., 2021).

Supplementary Movie 3: Whisker protraction leads to a convergence of the whisker search space with the visual space modulated by the whiskers themselves. Brighter red space: Modulated visual space extension caused by eye movements (± 20°). Darker red space: Modulated visual space. Turquoise whiskers indicate the ones identified to be important for tacto-visual integration in primary visual cortex (VISp). Whiskers were aligned to a realistic model of the mouse head (Bolanos et al., 2021).
